# Community Involvement in Response to Ebola Virus Disease Epidemic in North Kivu and Ituri, Democratic Republic of Congo: A Mixed-Methods Study

**DOI:** 10.29245/2578-3009/2023/S3.1110

**Published:** 2023-05-12

**Authors:** Tieman Diarra, Joseph Okeibunor, Amadou Baïlo DIALLO, Nkechi Onyeneho, Bairo Hamadou, Michel N’da Konan Yao, Zabulon Yoti, Ibrahima Socé FALL

**Affiliations:** 1 Independent Consultant, Mali; 2 World Health Organization; 3 University of Nigeria, Nsukka

**Keywords:** Community, Community members, Community leader, Ebola virus disease, Involvement, Participation, Information, Awareness, Capacity building

## Abstract

We investigated the involvement of community members in response to the Ebola Virus Disease (EVD) epidemic in the North Kivu and Ituri provinces of the Democratic Republic of Congo. This cross-sectional study, conducted using mixed methods of data collection, included a uniformly structured questionnaire survey, which was administered to 800 randomly selected adults (aged ≥ 18 years). Further, we used qualitative tools of inquiry—focus group discussions (FGD) and in-depth interviews (IDI)—to guide the context of the information collected in the survey. Community leaders, religious leaders, and Ebola survivors were interviewed using the IDI guide, while young men (≤ 30 years), young women (≤30 years), adult community males (<30 years), and adult community females (<30 years) were in separate FGD sessions. The results revealed that the urban area was the most affected by the epidemic (79.2%) compared to 20.8% in rural areas. The χ^2^ calculated was 18.183 (P<0.001). Community members exhibited varying degrees of involvement in response to the EVD epidemic in the two provinces. Community members were mostly engaged in information dissemination. However, they believe they could have contributed more if they had been fully engaged. These findings were derived from the qualitative data. The study contributes to evidence on how community involvement could help response to public health events globally, hence this study provides valuable insights for future public health interventions and response.

## Introduction

The response to the Ebola virus disease (EVD) epidemic has been organized by the national authorities of the Democratic Republic of Congo (DRC) and partners in the provinces of North Kivu and Ituri (International Federation of Red Cross, 2017). Community members also became involved in the fight in multiple ways as they were significantly affected by the disease, either directly or indirectly. Community involvement began with leaders, as they have been the main interlocutors of the response staff. These community leaders included village chiefs, neighborhood chiefs, street chiefs, avenue chiefs, and religious superiors of the Catholic and Islamic faiths. They undertook EVD control activities with the support of the response personnel and organized community meetings to inform and sensitize the population. In some cases, they assumed the responsibility of organizing inter-community meetings of village leaders.

Subsequently, other members of the community—such as religious people, teachers, traditional therapists, contacts, and family members—became involved in response to the epidemic. Churches, parishes, and mosques were used to spread awareness to religious people; hand-washing facilities were also installed in these places. The schools visited during this research had also been equipped with hand-washing facilities. In cases wherein religious people began disseminating discordant messages pertaining to the epidemic—messages fed by rumors about the disease—they ended up contributing to the response. However, this was rarely the case encountered throughout this research. Overall, religious people were more solicitous and involved in the response than traditional therapists^1^.

People from the communities, such as the local health personnel and town criers and members of civil society organizations, were also involved in activities to fight EVD. These people were trained by members of the response team and contributed to the fight against EVD in both urban and rural areas. Capacity building by response staff has promoted community involvement. The participation of community members was made possible by spreading information to the community and sensitizing them to the response activities. Several means were used to promote awareness, such as posters, the radio, and the media. Community members contributed to information, awareness, community-based surveillance, active case finding, contact tracing, and, in some cases, community watch. Involving the communities in these activities has been challenging^2^. The largest obstacle to ensuring participation in response activities was people’s reluctance to participate in them, which often led to insecurity^3–5^. However, community leaders have made it possible to reduce acts of violence against the response teams.

First, we examine the involvement of community members, civil society associations, and community leaders in the response. Thereafter, we discuss the role of village chiefs in managing insecurity. Finally, we describe the involvement of the religious people and the public, the difficulties of community involvement, and capacity building as the cement of community involvement in the response.

### Study Area

The study was conducted in the North Kivu and Ituri provinces, where residents are experiencing the 10th EVD outbreak in the Democratic Republic of Congo (DRC). **Ituri** is one of the 26 provinces of the Democratic Republic of the Congo. The capital of Ituri is the city of Bunia. Regional features include the Ituri Rainforest and high plateaus (2000–5000 meters), a large tropical forest, and savannah landscapes. The rainforest is located northeast of the Ituri River and on the western side of Lake Albert. Ituri is a region with rare fauna, including the okapi, the national animal of the Congo, and flora, including the Mangongo, an important tree species that produces the leaves that the Mbuti use to build their homes. The province’s population comprises primarily the Alur, Hema, Lendu, Ngiti, Bira, and Ndo-Okebo peoples, though figures differ as to which of the groups constitutes the largest percentage of the population. The Mbuti, a pygmy ethnic group, reside primarily in the Ituri forest near the Okapi Wildlife Reserve, though some Mbuti have been forced into urban areas by deforestation, overhunting, and violence. Additional features include the Kilo-Moto gold mines, which are partly located in Ituri, and petroleum reserves, which were found on the shores of Lake Albert by the Heritage Oil and Tullow Oil companies in the early years of the 21st century.

*North Kivu* (French: *Nord-Kivu*) is a province bordering Lake Kivu in the eastern Democratic Republic of the Congo, with Goma as its capital. North Kivu borders the provinces of Ituri to the north, Tshopo to the northwest, Maniema to the southwest, and South Kivu to the south. The eastern border is shared with the countries of Uganda and Rwanda. The province consists of three cities—Goma, Butembo, and Beni—and six territories—Beni, Lubero, Masisi, Rutshuru, Nyiragongo, and Walikale. The province is also home to the Virunga National Park, which is a World Heritage Site that is the home of the endangered mountain gorilla. Along with the heightened insecurity and isolation due to rebel activities, North Kivu is demographically like Ituri. However, this province is politically unstable and has been one of the flashpoints of the military conflicts in the region since 1998.

The ”2018 or 10^th^ Kivu EVD outbreak” began on 1 August 2018, when it was confirmed that four cases had tested positive for EVD in the eastern region of Kivu in the Democratic Republic of the Congo (DRC)^6–8^. The Kivu outbreak affected the Ituri Province; the first case therein was confirmed on 13 August^9^. This outbreak started just days after the end of the 2018 Équateur province Democratic Republic of the Congo EVD outbreak^10–11^.

The affected province and general area are currently undergoing a military conflict, which is hindering treatment and prevention efforts. The World Health Organization’s (WHO) Deputy Director-General for Emergency Preparedness and Response has described the combination of military conflict and civilian distress as a potential “perfect storm” that could lead to a rapid worsening of the outbreak^12^. Due to the deteriorating situation in North Kivu and surrounding areas, the World Health Organization, on September 27, 2019, raised national and regional level risk assessments from “high” to “very high”^12^.

### Study Design

This study was designed to explore and document experiences and lessons around the response to the 10^th^ EVD outbreak in the North Kivu and Ituri provinces of the Democratic Republic of Congo (DRC). We adopted a cross-sectional design with mixed methods of data collection. The design allowed for multiple windows of data harvesting; the mixed methods provide the benefits of both quantitative and qualitative approaches and guarantee the integrity and the robust interpretations and conclusions that this study topic and evaluation type merit.

### Selection of Study Population

The study population comprised adults aged ≥18 years living in the respective communities and response team members. A 2010 estimate put the population of North Kivu at 5,767,945. An estimate of the adult population at 70% of the total yielded an estimated 18 years and older population of 4,614,356. With an annual growth rate of 3.2%, the populations of North Kivu in 2019 were estimated as 7,658,406 (total population) and 5,360,884 (age ≥18 years population). For Ituri, the 2005 population was reported to be 4,037,561. The estimated age ≥18 years population, at 70% of the total, was 2,968,865. For 2019, Ituri population estimates were 6,275,305 for the total population (all ages) and 4,392,714 for those aged ≥18 years.

The EDV response teams in the two provinces included over 10,000 persons. The various response pillars included surveillance, risk communication, social anthropology, vaccination, infection prevention and control, treatment and care, safe and dignified burial, security, logistics, and administration, among others.

### Sample Size Estimation and Sampling Strategy

#### Sample size

This study was exploratory; however, to offer statistical conclusions about certain indicators of perceptions and practices juxtaposed with relevant demographic characteristics, we included a representative sample of the study population. Assuming a 50% chance of EVD control intervention acceptance at a confidence interval of 95% with an error margin of 5%, a sample size of 384 was calculated as appropriate for the quantitative study. Multiplied by two (for the two provinces), we needed a sample of 768 residents. We then rounded the figure up to 800 to allow some room for losses. The qualitative aspect of the study depended on the information saturation after initial pairs were collected from each respondent category.

#### Sampling Strategy

A multi-stage sampling technique was adopted in selecting the communities, households, and respondents in this study. Two administrative areas (that are epicenters of EVD outbreaks within each province) were purposively selected, and 10 communities were randomly selected from each of the two administrative areas in the province.

### Selection of the household and respondent

The center of the selected community was the reference point where the team spun a pencil to determine the first route and first household and, thereafter, move to the right to pick the next household and then continued until the number of households to be sampled was included. Where there was a *cul-de-sac*, the step was retraced, and a turn to the left and then to the right was made to continue the sampling process.

Once in a selected household, an adult (≥ 18 years) was randomly selected for inclusion as a participant in the study. The sex of the participants was carefully alternated. If a male was selected as the first household number, the focus for the next household was the selection of a female.

## Methods

The study was conducted using a mixed-methods approach with qualitative and quantitative techniques. The methodology for data gathering included in-depth interviews, focus group discussions, and surveys using a structured questionnaire. Thus, this type of study requires a strong focus on individual actors rather than state actors^13^.

### Techniques of data collection

#### Focus Groups

**Focus group discussions** (FGD): These were distributed as follows.

A set of questions covering different thematic areas were developed to guide the discussions. The questions covered health care services in the community, awareness of and practices for EVD, as well as assessment of the different pillars of the response interventions. For the FGDs, 8 to 12 individuals were selected for each session. A minimum of two FGDs were conducted in the selected communities, and separate male and female FGD sessions were held in each community. Overall, a total of 8 FGD sessions were conducted in each province.

#### *In-depth* Interviews

In-depth Interviews (IDI) were conducted in each community where FGD was carried out. The IDI was held with community/opinion leaders in the communities in the selected communities and team leaders of response pillars. Interviews were used to explore people’s opinions, views, and attitudes because practices, insights into the outbreak and response, and other socio-cultural factors could influence attitudes towards the response. The FGD guide was used for the in-depth interview, focusing on the thematic areas of interest to the evaluation.

#### Structured Questionnaire

A structured questionnaire was used for collecting quantitative data from households. The questionnaire addressed all the indicators that were used for answering the research questions. The questionnaire was structured with results from the qualitative study. It was categorized into the following sections: socio-demographic data, perception of health problems in the community, knowledge of EVD, perceived epidemiology of the disease in the communities, and sources of information about EVD. Others included issues on communication and community engagement, infection prevention and control in the communities, vaccination, and surveillance as well as treatment and care. Other sets of questions covered safe and dignified burial, psychosocial issues, logistic and security issues.

All interviews and discussions were tape-recorded, and detailed notes were recorded simultaneously, including verbal citations. Tape-recorded interviews were transcribed according to standard rules. Observations were also recorded and, together with the discussions and interviews, triangulated with the quantitative data to arrive at conclusions.

### Training and Pilot Trials

All instruments were ***translated*** into Swahili and French, the common languages spoken in the communities, and back translated to English for clarity of meaning. In each province, 10 Research assistants with substantial experience in interactive community research, the use of qualitative and quantitative techniques, and cultural sensibility were recruited and trained for three days in Beni and another three days in Bunia to ensure their knowledge of the study objectives and the use of the data collection instrument.

Training also included data entry into ATLAS.ti template (qualitative data) and EPI INFO (quantitative data). The instruments were reviewed after training for clarity, understanding, and sensitivity. Each province had a ***supervisor*** who worked with the Principal Investigator on data quality monitoring, safety advisory, and ethical conduct of the research, including the management of informed consent procedures. The study was conducted first in Ituri and then in North Kivu. The lessons learned from Ituri were used to manage the process in North Kivu, a more security- and logistics-challenged province. The ***data analyst*** developed and pre-tested the template for data entry and analysis using the pilot test output. Given the short period of the study, data were collected using pencil and paper instead of an android device. Fieldwork took 20 days in each province to complete before analysis and report writing.

### Data Management

All quantitative data were double-checked by the researcher before being entered for computer analysis. Data were entered into Epi Info and processed using SPSS. Descriptive statistics were used to determine the proportions of various categories of respondents and indicators and for comparison. Frequency tables and graphic illustrations were used for presenting the data.

Qualitative data consisting of FGDs, and In-depth interviews were transcribed from audio records to text. All textual data were analyzed using ATLAS.ti software package. Data were analyzed according to themes corresponding to the indicators in the quantitative data and triangulated during presentation to enable complementary and analogous interpretation. Given the continuous analytical process involved in qualitative analysis, the initial analysis of the key informant interviews and focus group discussions informed the final development of the structured questionnaire to be used in the study must be noted. This process further enhanced triangulation between the two sets of data to be collected. While the quantitative results provided us with statistical conclusions, the qualitative results emphasized what was said and provided illustrative quotes that contextualized and deepened the quantitative results.

### Ethical Considerations

The principle of do-no-harm was adhered to in the study. Informed study approval was obtained at the provincial, local administrative, community, and household levels; informed consent was obtained from all individuals that were involved in the study. The WHO/AFRO Ethics Review Committee provided ethical approval for the study, and all the researchers attended the mandatory training, which included substantial discussion of the ethical issues in research. Fifty percent of the research assistants were females, ensuring same-sex interviews and moderation of FGD sessions. The assistants were also trained and mandated to comply with child protection and gender sensitivity in the process of data collection and visits.

## Results

### Involvement of community members

Participants in the FGD sessions argued that involving communities begins by respecting the prevention measures they were informed about in the various awareness-raising activities. Community awareness was fostered in several ways. For instance, many community members have been involved in outreach activities. The chief of the Campement Pygmée village in the Mandima health area said:

After our sensitization sessions, all the members of the community commit to passing on information to each other, so everyone starts to understand, little by little.” Further, the chief of the village of Bamako mentioned that information was provided to the community members during a meeting with the public so that they could protect themselves against the disease and take appropriate safety measures. He also reported that information was provided during workshops.

Meanwhile, some community members played crucial roles in the *riposte* through involvement in community relays and other activities. The chief of Madududu village, in the Mandima health area, described their activities as follows:

We have community town criers who go from door to door to find out whether there are sick or deceased people. It is from them that we, the chiefs, are informed; we then call the riposte team to come assess the situation.

Furthermore, community members brought their relatives to the health center. As mentioned by the Madududu village chief: “*People who show symptoms of Ebola are taken to the health center*; *from there, we determine whether it is a suspect case. It’s at the general referral hospital in Mambasa. That’s where the Ebola Treatment Centre [ETC] is*.”

The chief of the Mambago district in Boikene also spoke about the involvement of the members of his community:

My community is involved in the activities to fight Ebola. They are sensitized and actively doing contact tracing. We are informed, and we control all the movements of people who arrive in our community because, as I told you, my neighborhood hosts all categories of displaced people.

Community members played a vital role in providing information about EVD. The chief of Butama village noted the following:

The community had taken the Ebola situation seriously. The problem was rather the lack of awareness. When the epidemic reached Mangina, it was the right time to sensitize Butama. When the epidemic reached our village, sensitization became difficult because the population was afraid. This is what caused them to revolt against the riposte team. This disease is caused by the lack of hygiene, then by the disregard of hygiene rules—and this is what made our village become affected. The severity of the situation is that if one person becomes affected and you are close to him or her, the disease can take you both down. Here, we have taken everything seriously, and we are engaged in the activities of the riposte.

**Figure 1:**
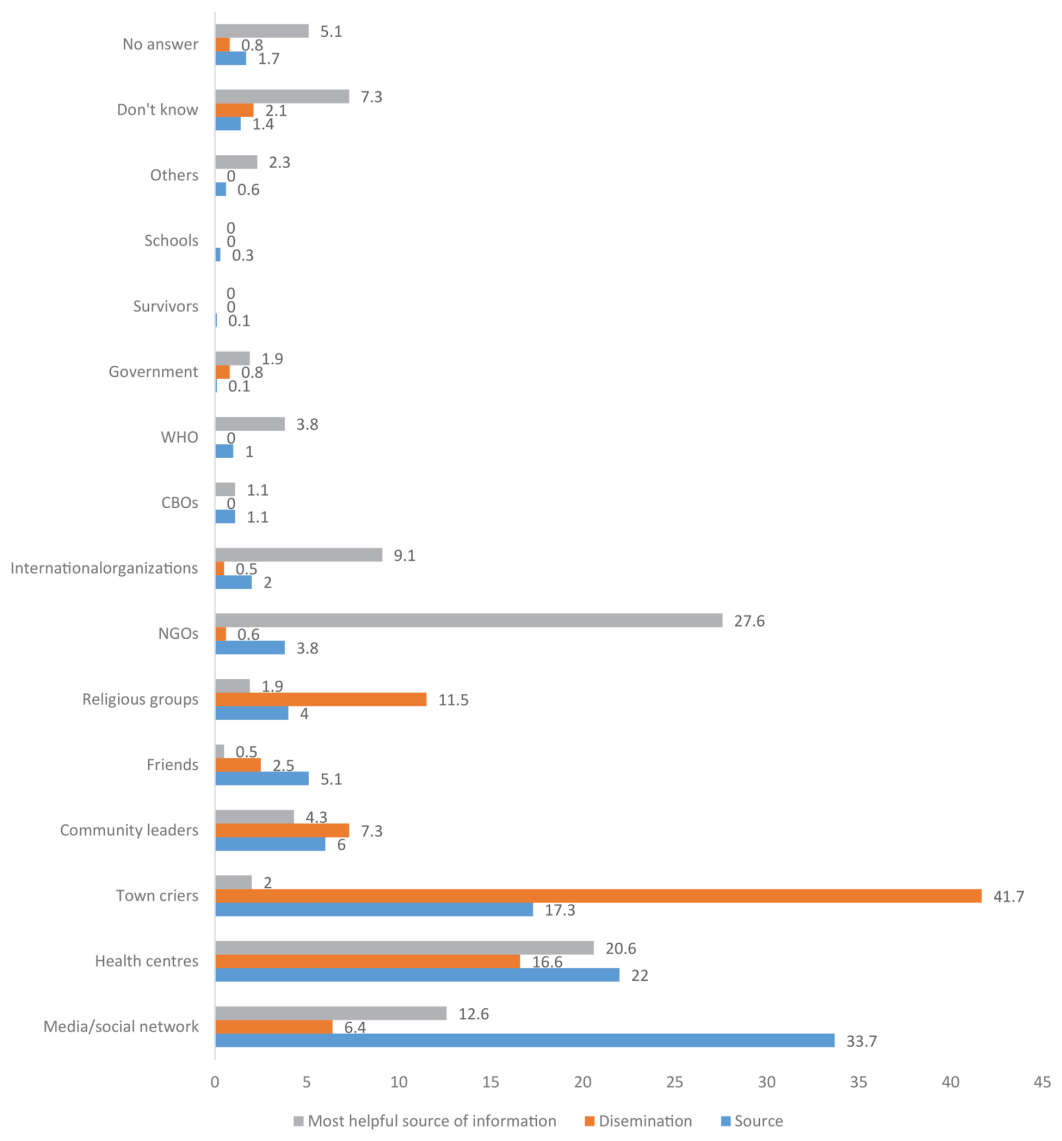
Sources of information on EVD in the communities and dissemination

The involvement of specific community members, such as village chiefs, neighborhood chiefs, avenue chiefs, other community leaders, religious people, and traditional therapists, was also examined in the quantitative study. The results are illustrated in Figure 1. The figure also lists the sources of information about EVD mentioned by the participants. One-third of the people surveyed received EVD-related information through media and social networks, approximately one-quarter received information through the health centers; fewer people reported receiving information predominantly from community relays and community leaders.

However, community members did not promote the idea that everything is performed within the support framework provided by other partners and people through the initiative of the State. This point raises the issue of visibility, which is discussed in the context of the operational EVD response. Community members spoke more about health agents’ workplaces than about the agents themselves. Only one participant reported a teacher as the person who provided them with EVD-related information, and one person reported a recovered person as their information source.

As for the dissemination of information regarding EVD, the situation is slightly different. Figure 1 also shows how such information was disseminated in the community, showing that a quarter of the people who were surveyed reported that information on EVD was disseminated through various community relays, followed by word of mouth among community members and health workers, then community leaders, media, and radio sources. Overall, 72% of the participants reported that EVD-related information was disseminated by one or some of the described sources. Finally, regarding the community relays, religious heads, and word of mouth, the religious heads and community health agents were the two main sources. Among all sources in the immediate proximity of the community members, information from friends and door-to-door campaign represented 94.6% of the sources of dissemination. However, information about EVD was disseminated in the community in different ways.

Figure 1 also lists those named by the participants as helpful in implementing preventive measures against EVD. Most people (over a quarter of participants) described NGOs as effective in implementing such measures, and more than a fifth mentioned health centers. Next, approximately 12.6% of the people mentioned that they had implemented the preventive measures themselves.

**Figure 2:**
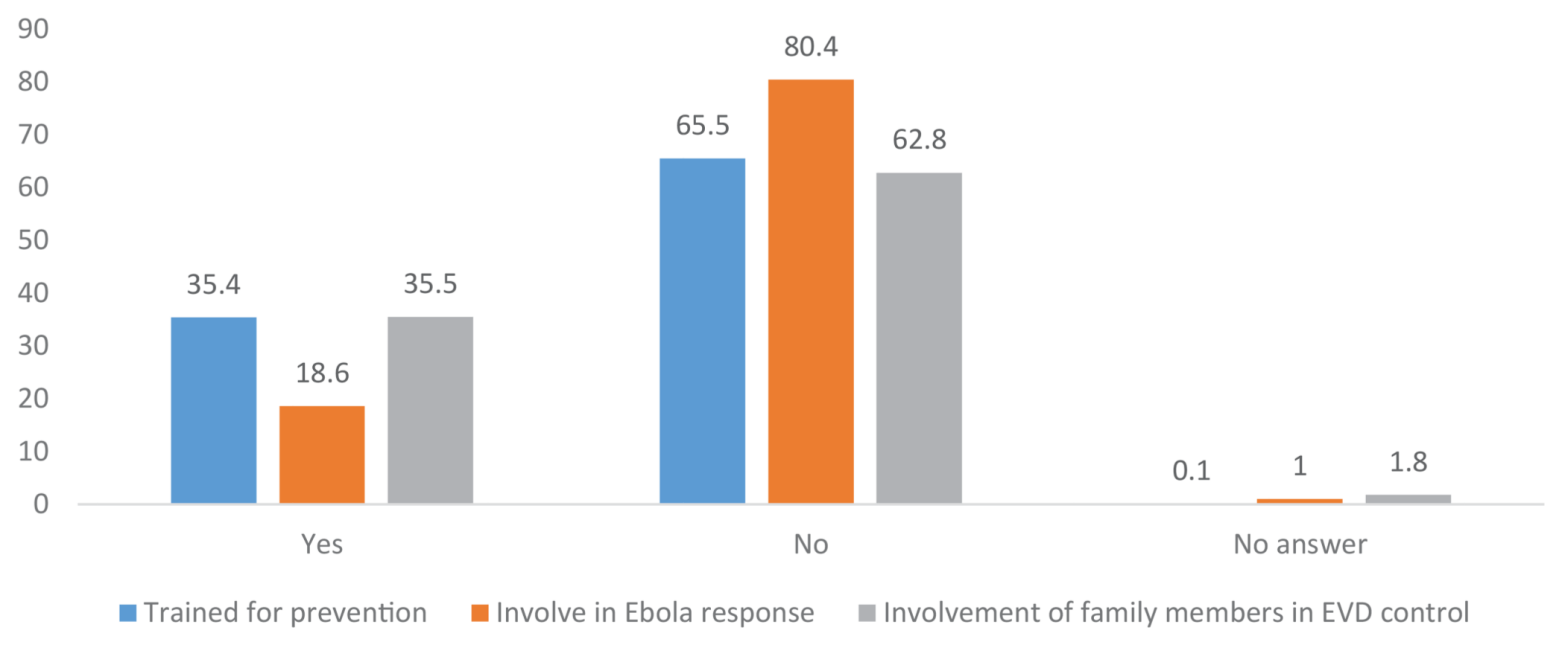
Community members trained for prevention and for EVD response and involvement of family members

At the operational level, the Congolese State, which is primarily responsible for the response, and the WHO, which plays a central role in coordinating the actions of partners, have been less visible in the communities. Community leaders, community relays, religious people, and community-based organizations were involved by the State and its partners, who were all cited more often. In the eyes of the communities, these are not commonly seen as sources working for the State or well-known partners. Meanwhile, community members involved themselves in the activities not only by disseminating information but also through training to prevent EVD, as presented in Figure 2.

More than one-third of the people surveyed had been trained to implement preventive measures against the EVD epidemic. This finding is a testimony to the great effort exerted to empower community members in the fight against the epidemic. The village leaders informed us about the instructions they received. This training, which focused on the involvement of community members in the *riposte* to the EVD epidemic, benefitted not only the community leaders but also religious people and traditional therapists. Indeed, community members were involved in active case finding and alerting, community-based surveillance, and disease prevention through the adoption of individual and collective measures. However, the training did not always lead to involvement in the response activities. Figure 2 shows that only 18.6% of people claimed to have been involved in activities to fight the EVD epidemic and that over one-third of the people surveyed had a family member involved in the EVD control activities. However, this involvement varies from one province to another, as shown in Table 1.

If family members of the people surveyed were involved in activities to fight EVD, most (60.2%) were in the North Kivu province; only 39.8% were in the Ituri province. The χ^2^ calculated is 24.528. The difference is significant at 0.001. This can be explained by the fact that North Kivu was the first one to be hit by the epidemic and the most affected. The situation also varied according to the setting, as explained below.

As presented in Table 1, respondents’ family members were more involved in the EVD epidemic control activities in urban areas than in rural areas. This was true in both provinces: 79.2% in urban areas versus 20.8% in rural areas. The χ^2^ calculated was 18.183. The difference is significant at 0.001. The urban area was the most affected by the epidemic. During research data collection, some rural communities had not been affected by the epidemic, while all urban communities were affected. Additionally, the involvement of a family member did not depend on the gender or educational level of the respondent.

**Figure 3:**
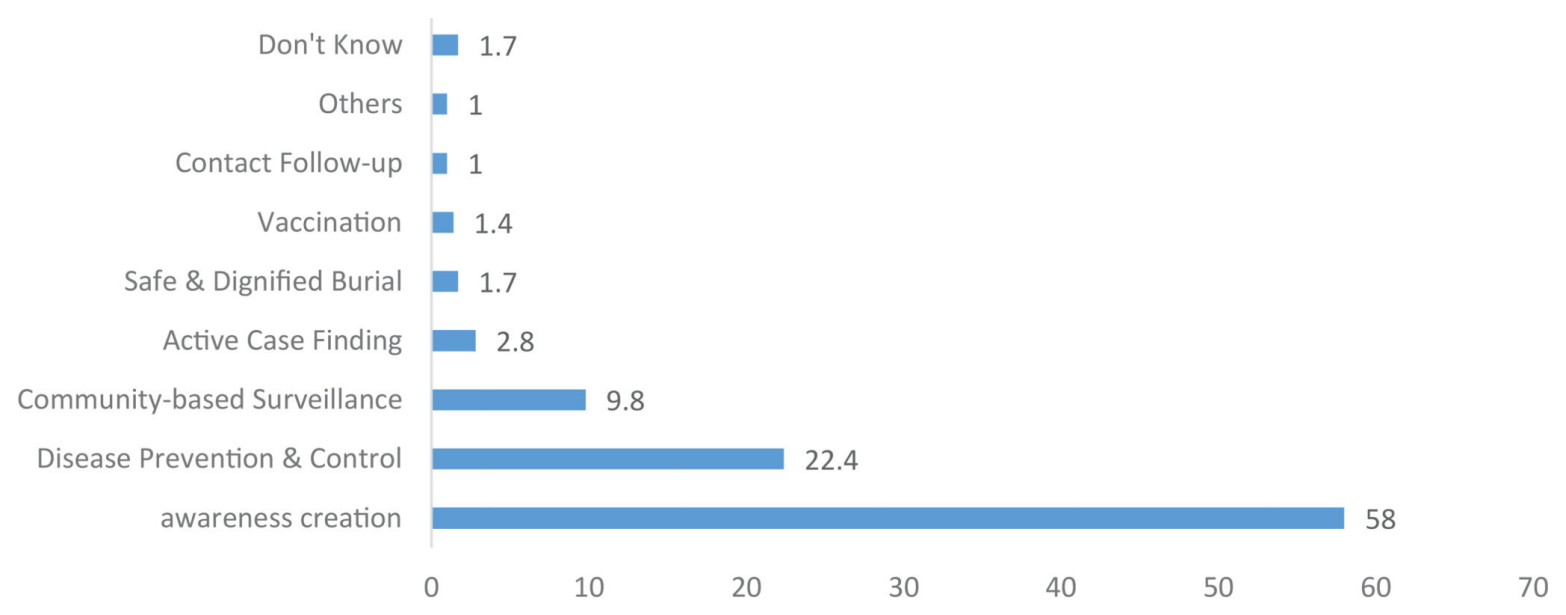
Different activities in which the family members of the respondents participated.

Figure 3 shows that more than fifty percent (58%) of the respondents indicated that their family members were involved in raising awareness among the citizens of the village or neighborhood (58%). Over one-fifth (22.4%) of the participants reported that their family members were involved in disease prevention and control activities. Participants also reported activities such as community-based surveillance, active search and alert, supervision, and follow-up of contacts.

## Discussion and Conclusion

The involvement of communities is essential to the implementation of health programs^2^. Numerous community members were involved in the response, including community relays, young people, family members, and the public. The village chief of Nyakunde, in the Mandima area, mentioned the work of young people in the fight against EVD in his community: “*We identify people who show symptoms of Ebola. If we find people with these conditions, we refer them to the health center*.”

Community members have played an important role in the response to the EVD outbreak in the provinces of North Kivu and Ituri. They have been involved in the *riposte* in various ways. First, community leaders—including village chiefs, neighborhood chiefs, street chiefs, avenue chiefs—and religious leaders of Catholic and Islamic faith have participated and introduced several response activities in the zones under their responsibility. They organized meetings to inform citizens and worked with staff involved in the response. Further, the response team often participated in the informative meetings organized by the local leaders. Moreover, religious leaders, teachers, traditional therapists, contacts, and family members were involved in the response within their communities. Religious people helped spread information about EVD in their churches, parishes, or mosques. Some of them diffused rumors about the disease, as was the case in Beni. They ended up supporting response activities. Similar points have been documented on other epidemics in the African Region. Studies have shown the significant roles of community members in dispelling rumors and misconceptions about the outbreak in Guinea (2; Boscarino et al., 2015).

Finally, members of civil associations and people involved with community resources—such as community health workers and community relays—were involved in activities to combat EVD. They worked in both rural and urban areas. These people were trained to enable them to assume such responsibilities. The involvement of the communities was facilitated by capacity building of its members by the staff involved in the *riposte*. Community members were identified and educated according to their responsibilities, either as community health personnel or community relays.

The participation of other community members was ensured through information and awareness-raising about *riposte* activities. The authorities in charge of the *riposte* at different levels provided information and awareness through varied means, including posters, the radio, and the media. However, some members of the community received information through social networks or from their neighbors. Some members conducted information-spreading and awareness-raising activities, while others were involved in community-based surveillance, active case finding, or contact monitoring. In some cases, they were involved in the community watch.

In certain villages, towns, or neighborhoods, the main issues that made community engagement difficult were residents’ reluctance to participate in *riposte* activities and insecurity. Community leaders have played a significant role in the implementation of *riposte* activities in peaceful settings. However, violence against the *riposte* teams has hindered and interrupted activities in some periods, although community leaders considered EVD a priority health problem.

## Figures and Tables

**Table 1:** Distribution of participants in the IDI and FGD sessions by provinces

Target	North Kivu Province				Ituri Province			
	Butembo		Beni		Mbuti		Bunia	
	IDI	FGD	IDI	FGD	IDI	FGD	IDI	FGD
Pillar leads	All		All		All		All	
Pillar members	2/pillar		2/pillar		2/pillar		2/pillar	
Community leaders^1^	≥2/ community		≥2/ community		≥2/ community		≥2/ community	
Leader of survivor group	≥2/ community		≥2/ community		≥2/ community		≥2/ community	
Community adult males		≥2 groups		≥2 groups		≥2 groups		≥2 groups
Community adult females		≥2 groups		≥2 groups		≥2 groups		≥2 groups
Community male youth		≥2 groups		≥2 groups		≥2 groups		≥2 groups
Community female youth		≥2 groups		≥2 groups		≥2 groups		≥2 groups
Survivors		≥2 groups		≥2 groups		≥2 groups		≥2 groups

1 Community leaders here include traditional, religious, political and social opinion leaders

**Table 2:** Involvement of family, village, or neighborhood members in the fight against EVD by setting and Province.

Perceived involvement	Province			Setting		
	Ituri	Kivu	Total	Rural	Urban	Total
Yes	113 (39.8)	171 (60.2)	284 (100)	59 (20.8)	225 (79.2)	284 (100)
No	284 (56.6)	218 (43.4)	502 (100)	178 (35.5)	324 (64.5)	502 (100)
No answer	3 (21.4)	11 (78.6)	24 (100)	4 (28.6)	10 (71.4)	14 (100)
Total	400 (100)	400 (100)	800 (100	241 (100)	559 (100	800 (100)

## Data Availability

The data that support the findings of this study are not publicly available due to their containing information that could compromise the privacy of the research participants. The data are available from the corresponding author (Joseph Okeibunor) upon reasonable request.
